# Coordination, Balance and Fine Motor Skills Deficities in Children with Autism Spectrum Disorder Without Co-Occuring Conditions—Application of MABC-2 Test in Pilot Study Among Polish Children

**DOI:** 10.3390/jcm14144946

**Published:** 2025-07-12

**Authors:** Katarzyna Stachura, Ewa Emich-Widera, Beata Kazek, Magdalena Stania

**Affiliations:** 1 Department of Health Sciences, Medical University of Silesia in Katowice, 40-752 Katowice, Poland; 2 FIZJOMED, 43-300 Bielsko-Biała, Poland; 3 Department of Child Neurology, Faculty of Medical Sciences, Medical University of Silesia in Katowice, 40-752 Katowice, Poland; 4 Child Development Support Center Persevere, Kępowa Street 56, 40-583 Katowice, Poland; 5 Institute of Sport Sciences, Academy of Physical Education, Mikołowska 72A, 40-065 Katowice, Poland

**Keywords:** autism spectrum disorders, motor disorders, dyspraxia, MABC-2 test, DCD

## Abstract

**Objectives**: The primary objective of this study was to determine whether motor disorders are significantly more prevalent in children with Autism Spectrum Disorder (ASD) without co-occurring genetic or neurological conditions compared to neurotypical children. Another aim was to explore the applicability of the MABC-2 test for assessing motor skills in a Polish cohort of children with ASD. Additionally, this study sought to develop a basic framework for motor skill assessment in children with autism. **Methods**: This study included 166 Caucasian children, both sexes, aged 5–12 years, without intellectual disability (IQ ≥ 70), without concomitant genetic or neurological disorders, particularly epilepsy or cerebral palsy. The study group consisted of children with ASD (n = 71), and the control group consisted of neurotypical children (n = 95). The participants were assessed with the Movement Assessment Battery for Children–second edition (MABC-2), MABC-2 checklist and the Developmental Coordination Disorder Questionnaire (DCDQ), used as a reference point. **Results**: The children with ASD obtained significantly lower MABC-2 test results in all subtests in comparison with the control group. The children with suspected or diagnosed coordination disorders were characterized by a significantly greater number of co-occurring non-motor factors than the other participants of this study. MABC-2 test showed greater consistency with DCDQ than with the MABC-2 questionnaire. **Conclusions**: Children with ASD present a lower level of manual dexterity and balance and greater difficulties in performing tasks, including throwing and catching, in comparison with neurotypical children. The MABC-2 test with the MABC-2 checklist and DCDQ questionnaire constitute a complementary diagnostic tool.

## 1. Introduction

The occurrence of deficits in communication and social interaction, as well as behavioral disorders, including narrow, focused interests and limited behavioral patterns, is characteristic of autism spectrum disorder (ASD). Autism spectrum disorder occurs throughout the entire world, regardless of socioeconomic status [1]. Data from the 2020 report of the Centers for Disease Control and Prevention in the USA indicate that 1 in 36 8-year-old children has been diagnosed with ASD [2]. Children with autism spectrum disorder are challenged with an array of different developmental and neuropsychiatric disorders that impact their daily functioning [3]. The simultaneous occurrences of epilepsy, cerebral palsy and intellectual disability are identified more often than within the general population [4,5,6]. The meta-analysis by Lai et al. [7], encompassing 96 research studies conducted between 1993 and 2019, demonstrated that ASD may be accompanied by ADHD, anxiety disorders, sleep disorders, behavioral disorders, depression, obsessive-compulsive disorder, bipolar disorder and schizophrenia. According to the literature data, sensory integration disorder (SI) occurs in 42–88% of people with ASD [8].

Apart from the barriers of communication and behavioral disorders, children with ASD present a variety of motor difficulties within the area of fine motor skills, which include all manual skills (often described as manual dexterity). The children who struggle with the problem of inadequate muscle tone cannot adjust pressure during writing and have difficulties with activities that require precision in the movements of the fingers and the opposable movements of the opposable thumb [9,10]. Motor deficits among children with ASD also affect gross motor skills relating to posture stability and eye–hand coordination. The results of the published research studies confirm the occurrence of postural defects, balance disorders, abnormal gait patterns and dyspraxia [11,12,13]. Coordination is closely related to balance and muscle tone, which include harmonious performance of full movement patterns while preserving an adequate muscle timing (the order of the tightening of muscle groups) and the stability of posture. Fluidity of movement depends on the processes of excitation and inhibition that take place in the central nervous system [14]. Somatognosia, praxis (planning and execution of movement) and adaptive abilities also affect coordination. School-age children with ASD have difficulties performing activities such as running, hopping, bouncing and kicking the ball or sidestepping [15]. In comparison with neurotypical children, they also need more time to perform rhythmical hand and body movement sequences [15,16]. People with autism have difficulties with accuracy when throwing a ball and maintaining stability of posture [16]. Performing tasks that require properly developed reactive balance, such as standing on one leg, riding a bicycle or scooter, or roller skating, often constitutes a considerable problem and may be the cause of exclusion from the peer group. Consequently, such situations lead to children giving up trying to make an effort and becoming reluctant to perform physical activities, which in turn promotes a sedentary lifestyle, obesity and the progression of behavioral problems of persons with ASD [17]. According to Ayres, approximately one-third of such persons have problems with adaptability as well as with learning self-service activities and thus require full-time care and assistance in everyday life [18].

The underlying mechanisms of motor and social communication impairments in ASD are both complex and multifactorial. Quantitative neuroimaging studies have revealed dysfunction-related abnormalities in several brain regions among individuals with ASD, including the cerebellum, brainstem and limbic structures [19]. Children on the autism spectrum frequently exhibit difficulties in integrating visual, vestibular and somatosensory inputs across multiple levels of the central nervous system [13]. As previously mentioned, they also commonly present with a range of motor impairments, such as atypical gait patterns and dyspraxia [11,12]. Motor skills—both gross and fine—play a critical role in social engagement by enabling participation in group activities and everyday play. Research indicates that autistic children with more pronounced motor difficulties often experience greater challenges in social communication [20].

Various assessment tools are commonly used to evaluate motor delays in individuals with ASD, such as the Bruininks–Oseretsky Test of Motor Proficiency and the Test of Gross Motor Development [21]. One of the recommended tests used to assess the level of motor functioning and the diagnosis of developmental coordination disorder (DCD) in children is the Movement Assessment Battery for Children—second edition [22]. Another well-researched and frequently used tool to diagnose DCD in children is the Developmental Coordination Disorder Questionnaire—DCDQ’07) [23]. Both of these tools were chosen as methods to assess the development of children, the results of which we hereby present.

The MABC-2 test is a commonly used tool by researchers to detect motor disorders. In Poland, no studies have yet been conducted using this tool. There are also a few reports on balance, coordination and fine motor skills in a homogeneous group of patients with ASD; for example, those without intellectual disabilities. Scientific reports, especially in recent years, indicate that among children with ASD, the problem of impaired coordination translates into difficulties in social functioning, low self-esteem and avoidance of physical activity. The issue of lack of physical activity among children and adolescents with ASD is not widely recognized, and objective methods for assessing motor skills are not sufficiently widespread. We hypothesized that the MABC-2 test, combined with the MABC-2 checklist and the DCDQ questionnaire, constitutes an optimal diagnostic tool for assessing motor skills in children with ASD. The use of objective tools for assessing coordination in children in rehabilitation clinics, physiotherapy offices and educational institutions would enable reliable diagnosis of disorders, evaluation of therapeutic progress and interdisciplinary planning for the improvement of children with ASD.

This study is the first to utilize the MABC-2 in a Polish cohort of children with ASD, thereby contributing to the limited regional data available. The primary aim was to determine whether impairments in balance, eye–hand coordination and manual dexterity are significantly more prevalent in children with ASD compared to their neurotypical peers. Additionally, this study explored the applicability of the MABC-2 test for assessing motor skills in this population. A further objective was to establish a basic framework for evaluating motor coordination in children, with particular emphasis on those diagnosed with ASD.

## 2. Materials and Methods

This study was carried out between May 2020 and December 2021. The research protocol was approved by an ethics committee of the Institutional Review Board, Medical University of Silesia, Katowice, Poland (PCN/CBN/0052/KB1/31/I/20/22). All parents gave informed written consent for their child’s participation.

### 2.1. Participants

A total of 166 Caucasian children of both sexes, between the ages of 5 and 12, qualified for this study. The children were divided into two groups, the study group (n = 71) and the control group (n = 95). The study group consisted of children remaining under the care of the Child Development Support Center “Persevere” in Katowice, as well as children whose parents learned about the research project through the information published in social media.

In order to ensure the highest homogeneity of the study group, the following criteria were applied in this study: the presence of autism spectrum disorder identified by an ASD psychiatrist using the “gold standard” diagnostic test, the ADOS-2 [24], as well as criteria consistent with the DSM-V classification; the ability to understand commands and communicate; the skills to perform the gross and fine motor tasks. The exclusion criteria included the occurrence of intellectual disability (IQ ≤ 70), genetic and neurological disorders (including epilepsy and idiopathic cerebral palsy). Children were consulted by a child neurologist who, along with the physiotherapist, carried out the qualification process for participation in the research study.

Children without any autistic characteristics or intellectual disability (IQ ≤ 70), any other known genetic and neurological disorders, including epilepsy and idiopathic cerebral palsy, qualified for the control group.

All children participating in the research study, conducted with the MABC-2 test, completed the entire battery of trials. There was no need to exclude any of the qualified participants or reschedule any of the trials.

**Figure 1:**
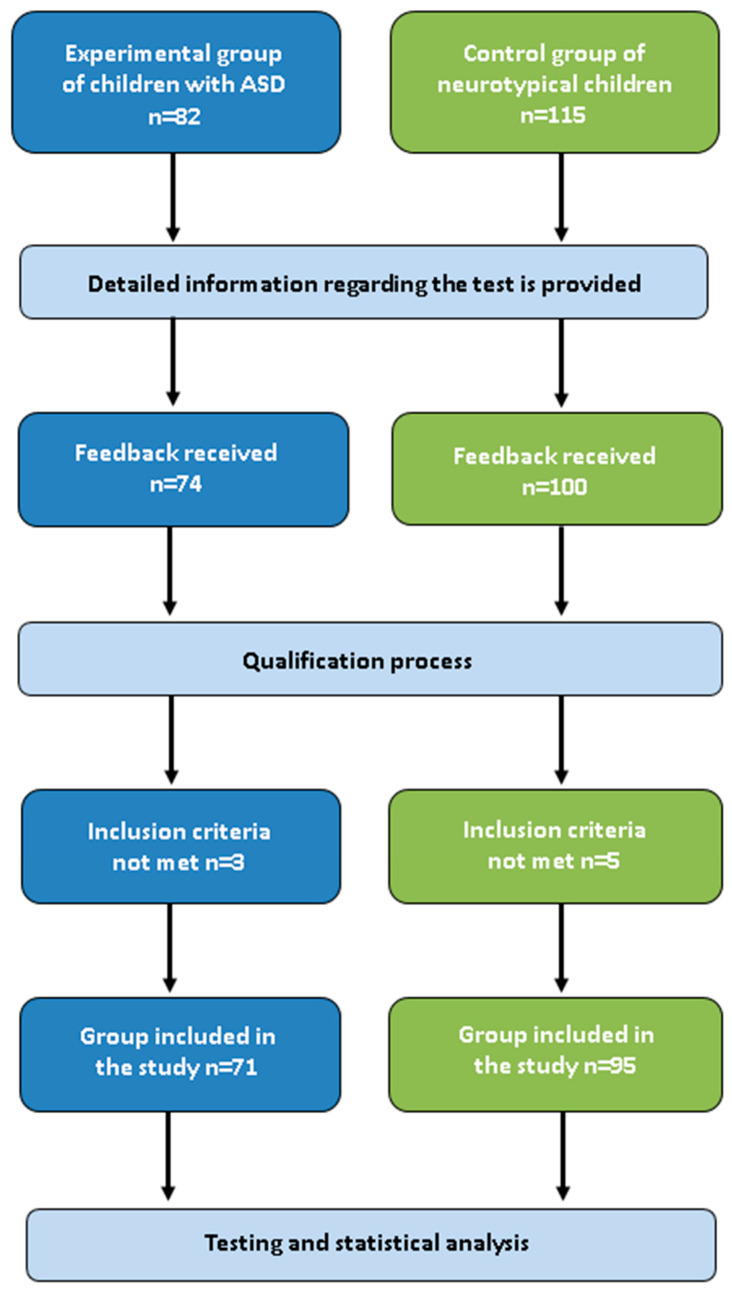
Outline of the qualification process to this study.

Eight children were excluded as they did not meet the inclusion criteria. Ultimately, the study group consisted of 71 children with ASD, and the control group consisted of 95 neurotypical children. The qualification process for the research study is presented in the following illustration (Figure 1).

The groups did not differ significantly in relation to age, weight, height and body mass index (BMI). The statistically significant differences were noted only by comparing the groups by sex. The number of boys among the children with ASD was higher than among the children in the control group; respectively, 66.20% and 49.47%. The median age of the study group of 166 participants was 9 years of age.

### 2.2. Assessment

This study was conducted at the Child Development Support Center—PERSEVERE, Katowice, and at a primary school in Mikołów, Poland. The assessments were carried out in quiet, child-friendly rooms to minimize sensory overload and other environmental distractions.

Motor development was assessed using the Movement Assessment Battery for Children–second edition [25], along with MABC-2 checklist and the DCDQ’07 questionnaire [26].

MABC-2 is used to assess the motor development in children between the ages of 3 and 16. It has been applied across a range of populations, including those with DCD, ADHD, intellectual disability, hearing impairment, prematurity and ASD [27,28,29,30,31,32,33,34,35,36,37,38]. Studies from various countries confirm its high reliability, diagnostic and prognostic validity and internal consistency (ICC = 0.78–0.96 overall; sensitivity 70–90%) [39,40,41,42,43]. MABC-2 comprises eight movement tasks, which are divided into three categories: manual dexterity, skills related to throwing a ball, such as aiming and catching (AC) and balance (BAL). The exercises are divided into three age groups: I. 3–6; II. 7–10; III. 11–16. The tasks are varied and their level of difficulty is adapted to the children’s age. The sex-related differences are not factored in. The raw results are calculated according to the instructions for the standardized results. The total amount of the achieved standard points is recalculated into percentiles. The lighting signaling system is utilized to interpret the results. The scoring between 57 and 67 (6th–15th percentile)—yellow color—indicates the possibility of the occurrence of the motor difficulties. The test tasks were implemented in the order recommended by the authors of the test [25]. A child would first familiarize itself with each task using illustrations, oral instructions and demonstrations. The time to perform the test was between 30 and 40 min. All children with ASD performed the test in the presence of their parents or caregivers, who were not allowed to provide them with any instructions or speak with them during the test. The test was conducted by a physiotherapist with a Master’s degree and ten years of practice with children, including children on the autism spectrum.

The Developmental Coordination Disorder Questionnaire (DCDQ’07) is widely recognized as a reliable tool in identifying motor impairments. The DCDQ should be filled out by a parent or a legal guardian of the child. It is designed for diagnosing DCD in children aged 5 to 15 years. The DCDQ consists of 15 questions, rated on a five-point Likert scale. Completing the questionnaire takes 10–15 min. The total score, derived from the points assigned to the answers to all questions, ranges from 15 to 75 points. The results are interpreted for three age groups (I: 5 years to 7 years and 11 months; II: 8 years to 9 years and 11 months; III: 10 years to 15 years).

The MABC-2 questionnaire is an integral part of the MABC motor test. Thirty questions were divided into part “A”—movement in a predictable, static environment, and part “B”—movement in an unpredictable, dynamic environment. Results: 85th–94th percentile (inclusive) suggest further observation of the child and the possibility of the occurrence of motor impairments. Additionally, the questionnaire allows for the collection of information regarding non-motor factors that affect movement. The questionnaire takes 10 min to finish. The MABC-2 questionnaire and DCDQ were filled out by the parents or the legal guardians.

### 2.3. Statistical Analysis

Excel 2007 and STATISTICA v12 software suite were utilized in the statistical analysis of the achieved results. The analysis of the distribution of quantities was conducted utilizing the Shapiro–Wilk test, which indicated statistically significant differences between these distributions and the hypothetical normal distribution. In the descriptive statistics, a median (Mdn) was used as the positional parameter, and a quartile range was provided to determine variables. The nonparametric Mann–Whitney U test was used in the statistical calculations of the differences in the results between two groups. In cases where a statistically significant difference was obtained in a statistical test, effect size measures were calculated: *r*(*G*)—glass rank biserial correlation coefficient for the Mann–Whitney U test (threshold values: 0.10—small effect, 0.30—medium effect, 0.50—large effect, 0.70—very large effect), *ε*^2^—epsilon squared coefficient (threshold values: 0.01—small effect, 0.06—moderate effect, 0.14—large effect). The proportions (fractions) of the occurrence of the particular categories were used to describe the nominal (discrete) quantities, and the CHI^2^ Test with the Yates Continuity Correction was used in analyzing the differences between the groups. The correlation of quantitative variables was performed based on the Spearman test, providing the correlation coefficient and the result of the significance test. To assess the reliability of the questionnaires used in this study, the Cronbach’s α coefficient was calculated. In all tests, the level of statistical significance was set at *p* ≤ 0.05.

## 3. Results

### 3.1. MABC-2 Test Results

The analysis of the results indicated that the children in the study group achieved lower scores than the children in the control group in all three components of the MABC-2 test (Table 1). The median of the total standard score that was achieved by the children with ASD was 8 points (Mdn = 8), and 13 points were achieved by the children in the control group (Mdn = 13). The median of the test results in percentiles was 25, and in the control group, it was 84.

In the groups divided by age, children with ASD in the II age group achieved the highest standard scores in the manual dexterity category (Mdn = 9) and balance (Mdn = 9.5), while children in the I age group performed best in tasks assessing aiming and catching (Mdn = 11). Comparison of the study and control groups, taking into account the age intervals, revealed significant differences in all categories except for balance in the III age group (Table 2).

The total result in percentiles for the I, II and III age groups was equal to, respectively, Mdn_I_ = 25; Mdn_II_ = 31; Mdn_III_ = 20.5 in the study group and Mdn_I_ = 84; Mdn_II_ = 84; Mdn_III_ = 84 in the control group.

To the red zone—indicating the presence of coordination disorders—18 children with ASD (25% of the group) and 2 children from the control group (2%) were classified. In the yellow zone, indicating a high risk of disorders, there were 13 children from the study group (18%) and 2 from the control group (2%).

### 3.2. MABC-2 Check List Results

Cronbach’s α reliability coefficient indicated internal consistency of the entire MABC-2 questionnaire (α-Cronbach = 0.8). The children from the study group achieved a statistically significantly higher score in comparison with the control group. The median of the total score of the MABC-2 questionnaire in the study group was 32 (Q_1_ = 22; Q_3_ = 49), while in the control group it was equal to 6 (Q_1_= 2; Q_3_ = 14). Through the use of the MABC-2 questionnaire, a high probability of the occurrence of motor impairments was discovered in 62 children with ASD and in 14 children of the control group.

Two children with ASD (2.82% of the group) and 14 children from the control group (14.74%) were classified into the yellow zone, where further observation for the potential occurrence of motor coordination disorder is advised.

### 3.3. DCDQ Questionnaire Results

The values of Cronbach’s α coefficient indicated internal consistency of the entire DCDQ questionnaire (α-Cronbach = 0.7). Children in the study group achieved a statistically significantly lower score in DCDQ (Mdn = 48; Q_1_ = 39; Q_2_= 56) in comparison to the children in the control group (Mdn = 66; Q_1_ = 61; Q_2_ = 71). Based on the DCDQ questionnaire, the occurrence of coordination disorder was suspected in 48 children with ASD (67.61%) and in 8 children from the control group (8.42%). The differences between the groups were statistically significant.

### 3.4. Consistency of Results of MABC-2 Test, MABC-2 Check List and DCDQ Questionnaire

MABC-2 test indicated a greater consistency of the DCDQ questionnaire (82,53%) assessments than of the MABC-2 checklist (64.46%). The result correlation test of the MABC-2 test and the DCDQ questionnaire, conducted with Spearman’s test, indicated a strong correlation in the study group (R = 0.544) and an average correlation in the control group (R = 0.390).

The analysis of the association of the total scores of the DCDQ questionnaire and the MABC-2 checklist indicated a strong negative correlation both in the group with children with ASD (R = −0.543) and in the control group (R = −0.635). The negative correlation is related to the fact that a high score on the DCDQ means a lower risk of the occurrence of motor impairments, and the higher scores on the MABC-2 checklist are related to a higher probability of the occurrence of the coordination disorder. No statistically significant correlation was observed between the MABC-2 test and the MABC-2 checklist in the control group.

### 3.5. Non-Motor Factors in Children with Suspicion or Occurrence of Coordination Disorder

Children whose results from the MABC-2 questionnaire suggested the suspicion or the occurrence of the coordination disorder were observed to have the non-motor symptoms, such as disorganization, forgetfulness, inattentiveness, impulsiveness, hyperactivity and underestimating own capabilities (Table 3).

The median of the number of the non-motor symptoms among the children with the suspicion of the occurrence of the coordination disorder was higher than among the rest of the participants of this study: Mdn = 9 (Q_1_ = 7; Q_3_ = 10) for MABC-2 test; Mdn = 8 (Q_1_ = 6; Q_3_ = 9) for MABC-2 checklist and Mdn = 8 (Q_1_ = 7; Q_3_ = 10) for DCDQ questionnaire. Among the children without the risk for the occurrence of the disorder, the median of the number of the non-motor symptoms was Mdn = 6 (Q_1_ = 5; Q_3_ = 9) for MABC-2 test; Mdn = 6 (Q_1_ = 3; Q_3_ = 7) for MABC-2 checklist and Mdn = 6 (Q_1_ = 4; Q_3_ = 8) for DCDQ questionnaire.

### 3.6. Basic Motor Assessment Scheme for Children

**Figure 2:**
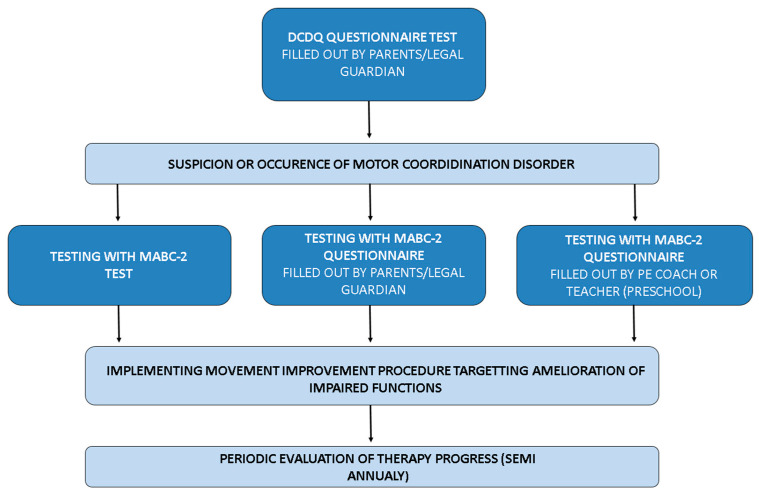
Basic framework for motor assessment in children with ASD.

One of the planned results of the implemented research study was creating a basic motor coordination assessment scheme for children, especially for children with ASD (Figure 2).

## 4. Discussion

In general, research studies assessing motor skills in children with ASD are not numerous and are frequently conducted on small sample groups [44,45,46,47,48,49,50,51,52]. Results achieved in our study show that motor impairments may constitute a significant phenotype characteristic of children with ASD without co-occurring conditions. Concentrating on this group of children seems reasonable, because, although, of course, the help and the broad support are generally offered to people with ASD, it is the persons without intellectual disability who have potentially considerable chances for relatively independent functioning and integrating with neurotypical peers. In this aspect, having good motor skills is very important. Children with autism spectrum disorder who go to regular schools confront healthy children in activities during physical education (PE) classes, during breaks or in the after-school programs for which good motor skills and coordination are the basic requirement to be part of the group. Motor impairments may be the cause for exclusion, the deterioration of general well-being, difficulties in learning, avoiding physical exercise and, as a result, they may lead to the exacerbation of behavioral and non-mental issues, such as obesity.

The MABC-2 test is the edited version of the study tool that was developed earlier (Henderson and Sugden 2007), which was validated based on the studies conducted in Canada, the United States of America and Great Britain [53]. Next, it was translated into many European languages, as well as Chinese [38]. It was used by researchers in different countries and cultural contexts; for example, in the Czech Republic, Sweden, Japan, Holland, Hong Kong, Israel, the United States of America and Greece, among others [27,44,54,55,56,57]. MABC-2 is currently one of the most frequently utilized tools in assessing motor impairments in children.

Children with ASD achieved significantly lower scores in comparison with the control group in all three categories in the test conducted by the authors of this research study. The small sample size does not allow for the generalization of the obtained results to the population of children with ASD without comorbid neurological and genetic disorders. However, demonstrating a significant difference in motor skills between children with ASD and neurotypical children in this pilot study was intended by the authors to serve as a foundation for validating the MABC-2 test in Polish children. The obtained effect size values are high, which supports the existence of a relationship between the observed differences in motor performance and autism. If these findings were confirmed in large samples tested with the MABC-2 validated in Poland, they would carry substantial clinical significance.

Odeh at al. [38] achieved similar results in assessing manual dexterity, aiming and throwing and balance among twenty four neurotypical children and children with autism spectrum disorder, aged between 5 and 12. In his research study, the average of the total result of the MABC-2 test in the group of children with ASD was x = 4.67, while in the group of neurotypical children, it was x = 8.17 [38]. In the research study conducted by Ament et al., the results of the MABC-2 test conducted among 56 children with ASD (without intellectual disability) were compared with the results of 81 neurotypical children and 63 children with ADHD [16]. The level of motor skills was significantly lower in the group of children with ASD than in the group of neurotypical children. Some researchers suggest that children with ASD may not understand the oral commands during assessments conducted with the help of motor tests. The incorrect interpretation of a command, and not the actual motor skills, may affect the achieved results. A study where the Test of Gross Motor Development-2 (TGMD-2) was utilized indicated that the participants did not understand the differences between sentences (e.g., between throwing low and throwing high) and required additional, individualized instructions or help in order to execute the commands [58]. Liu and Breslin [59] checked the result of using illustrated instructions for the test tasks of MABC-2. Children with ASD for whom the tasks were presented in pictures (without any particular oral instruction) achieved significantly higher scores (60% in the red zone; 16% in the yellow zone) than those children for whom elaborate oral instructions together with a demonstration, and without the presentation of illustrations of the exercises, were provided (96% in red zone). Considering the conclusions from the presented study, each exercise was described with a short oral comment, presented in pictures included in the MABC-2 instruction manual, as well as demonstrated in a physical form in order to allow for the best understanding of the commands and consequently facilitate the achievement of higher scores than the one achieved in the abovementioned study of Liu and Breslin [59].

The regulation of motor activities such as maintaining balance, throwing and catching is a complex process involving both the nervous and musculoskeletal systems [60]. Postural control, whether during quiet standing or dynamic movement, depends on the integration of sensory input from the vestibular, visual, proprioceptive and tactile systems. The cerebellum plays a crucial role in coordinating movement and ensuring the precision of motor actions by processing sensory information [61]. Neuroimaging studies have identified functional abnormalities in various brain regions in individuals with ASD [19]. Moreover, individuals with autism exhibit deficits in the sensorimotor integration of visual, vestibular and somatosensory stimuli at different levels of the nervous system [13], which may negatively affect postural control and overall motor performance. Difficulties in motor planning (apraxia) and impairments in executive motor functions further exacerbate these challenges [11]. Our study supports these findings, revealing that children with ASD scored significantly lower than their typically developing peers across all components of the MABC-2: manual dexterity, aiming and catching, and balance.

Associations between nervous system development and motor skills have been observed in individuals with neurodevelopmental conditions [62]. It has been suggested that the period between 7 and 8 years of age is crucial for the development of postural control [63]. Children under the age of 7 often struggle to use sensory information to estimate body position in relation to overall body dynamics [64], which is confirmed by our findings—children with ASD aged 5–6 achieved the lowest standard scores in the balance category. In each age group, typically developing children scored significantly higher than children with ASD in the respective MABC-2 test categories. The only exception was balance in children aged 11–12, where no significant differences were observed. This may be due to the fact that older children with ASD often attend sensory integration therapy or other interventions, which can enhance their balance abilities.

The issue of gender imbalance in the study groups reflects the pronounced male predominance in autism prevalence. This is particularly evident in cases of autism without co-occurring neurological or genetic conditions—so-called “pure autism”—which is becoming increasingly apparent in older age groups of children and adolescents with ASD.

The results gained by Liu and Breslin, as well as Belaire et al., do not indicate the significant influence of the sex type on coordination among people with ASD [37,59]. Engel-Yeger et al., as well as Kita et al., came to different conclusions [55,65]. Some researchers claim that the differences in the levels of motor skills may be caused by the social and environmental factors more than by the biological factors, especially among school children prior to the puberty period [66]. In our study group, no significant differences depending on the sex type were identified. Even though girls achieved better results in manual dexterity and balance, and boys showed better performance in tasks assessing catching and aiming, the differences were not statistically significant.

Liu and Breslin indicated the occurrence of delays in motor development in 80% (n = 24) of children with autism [59]. In a different study, 51 children with ASD between the ages of 7–12 were tested. Eighty-two percent of the participants were qualified for the red zone, and the risk of the occurrence of motor impairments was indicated in 8% of the participants [67]. In our study group, on the other hand, the percentage of children with ASD, in which considerable problems with coordination were indicated, was lower and was equal to 43%. A total of 25% of children were diagnosed in the red zone, and 18% of children in the yellow zone. The difference in the results may come from the use of different inclusion criteria in the research study. In the abovementioned publications [59,67], the information regarding the co-occurrence of intellectual disability or other genetic and neurological diseases in the tested persons was not provided, while that was the exclusion criterion in the experiment conducted by the authors of this study.

The MABC-2 checklist constitutes a valuable tool in fulfilling the diagnosis and describing children with the suspicion of motor impairments in detail. A reliable assessment requires, nevertheless, substantial knowledge of the child’s competencies from the person undertaking this task of filling out the questionnaire. Due to the characteristic outline of the checklist and the division of the self-service activities at home and the skills used at school, the assessment of a child’s motor skills may differ depending on the group of respondents. Reports in the literature confirm a significant influence of the person filling out the questionnaire on the final result. The research study conducted by Junaid [68] on 47 school-age children, between the ages of 7 and 8, where the results of MABC-2 questionnaires filled out by schoolteachers and physical education coaches were compared, indicated that the method of viewing motor skills is different depending on the group performing the assessment. The results of that study indicated that the physical education coaches had greater knowledge regarding motor skills than the children’s teachers [68]. In yet another experiment [43], the MABC-2 checklist results were also compared, factoring in dividing the respondents into groups. One group consisted of parents of 40 neurotypical children between the ages of 7 and 10, the second group consisted of teachers, and the third group consisted of physical education coaches. The results of the questionnaires achieved in the groups of teachers and parents did not differ significantly. The comparison of the scores between the groups of parents and physical education coaches indicated significant differences. Parents rated their children’s motor skills more critically (42.5% in red zone, 15% in yellow zone) than PE coaches (5.3% red zone, 16% yellow zone) [43]. In the research study we conducted, the tendency to rate the skills among the children with ASD lower was also observed among the parents and legal guardians. Due to the risk of having falsely positive indications of motor impairments (red zone), the MABC-2 questionnaire should absolutely be treated as a complementary tool together with the MABC-2 Test.

The application of the checklist in this study helped reveal the occurrence of significant motor impairments in n-62 children with ASD (87.62% of the group). Two children with ASD (2.82%) qualified for the yellow zone, for which further observation towards the possibility of the occurrence of the impairments is advised. Similar results were achieved by Belaire et al. in a group of 28 children with ASD, where the researchers, using the MABC-2 checklist, qualified 92% of the participants to the red zone and 4% to the yellow zone [37]. A research study conducted by Barnett and Henderson included 20 children with developmental coordination disorder between the ages of 6 and 11 [54]. With the help of the light signaling system of the MABC-2 checklist, it was indicated that 19 children with diagnosed coordination disorders had significant motor impairments. Another study conducted on 24 boys with Asperger’s Syndrome [69] indicated that motor impairments occurred in 23 children. The MABC-2 questionnaire may constitute a screening tool for the purpose of identifying any general motor difficulties to allow for further detailed diagnosis of the disorder by using the MABC-2 motor test. The good internal consistency of the MABC-2 checklist has been described in the literature [42] and was confirmed in our own studies with the use of Cronbach’s α test (0.822; 0.884).

It is worth mentioning that the consistency of the MABC-2 test with the MABC-2 checklist or DCDQ questionnaire is reviewed differently. In Shoemaker’s study, the greater concurrence of results of the motor test with MABC-2 questionnaire was noted to be 80%, while our study indicated greater concurrence with DCDQ (82.53%) than with the checklist (64.46%) [42]. The correlation between the MABC-2 test and questionnaire that was identified by us (R = 0.288) is lower than the one identified in the study of Shoemaker et al. (R = 0.38) as well as in the standardization trial in Great Britain (R = 0.55) [42,58]. The results of our studies as well as the results of Najfabadi (R = 0.60) and Ray-Kaeser (R = 0.80) indicated high correlation between the Motor Test and DCDQ (R = 0.54) [70,71]. It is worth noting that DCDQ questionnaire is commonly known and is highly regarded as a research tool, while MABC-2 checklist becomes a wholesome tool when put together with the test, which in turn provides more detailed information about the tested person than DCDQ questionnaire.

Among the children with ASD and the control group, a percentage of persons qualified to the yellow zone is small; therefore, we may speculate that the first worrisome symptoms of motor impairments have been overlooked. According to Blank et al. [23], the nowadays available motor skill tests have sensitivity of below 90%. This means that about 10% of children with a significant coordination disorder remain unidentified during diagnosis with tests such as, for example, MABC-2 or BOT-2. It is officially recommended to acknowledge the 16th percentile to be the cutting point for the total result (standard result ≤ 7), and the results at the level of at least the 5th percentile should be treated as unequivocal proof of coordination disorder, as long as the child meets all the other criteria [23]. If there are any clear reasons indicating a greater risk of motor impairments, and the results of one standardized motor test are above the determined cut off criteria, the test should be repeated by another researcher using MABC-2 test or a different battery of motor assessment.

Although questionnaires undoubtedly provide valuable information, they do not always reflect the real skills of a child. On the other hand, the results of the motor test may affect certain other aspects, such as the child’s level of anxiety, distraction by the new situation or relation with the person performing the assessment. It is then reasonable to use the motor test as well as the assessments based on the opinion of the parents or teachers during the diagnosis. The MABC-2 test, which is an objective, standardized tool, together with MABC-2 and DCDQ questionnaires, constitutes a complementary set providing information about the motor skills, which should be objectively and regularly assessed [23,26,42]. Tests assessing the efficacy of the physiotherapeutic interventions would have significant meaning in facilitating the development of therapeutic approach models oriented towards the improvement of coordination of the children with ASD [72]. It is worth noting that all children with autism spectrum disorder require the support of different therapists, especially including physiotherapeutic amelioration, in order to provide proper motor development and facilitate the performance of daily activities without problems and the best possible functioning in society [73].

Despite certain limitations, this study has strengths that give it cognitive and practical value. First of all, it concerns a topic that is not often addressed—the specificity of motor disorders in children with ASD without co-occurring neurological or genetic disorders. Such a narrowing of the research group allows for a better understanding of the autism spectrum itself, without interference resulting from other disease entities. The practical approach is also important—a comparison of the MABC-2 test tools and the DCDQ questionnaire allows for determining their diagnostic usefulness, which can have real application in education and therapy. Additionally, indicating the relationships between motor difficulties and other factors (non-motor) creates a basis for further, more complex analyses. Finally, the work contributes to the development of a diagnostic framework and provides a foundation for the development of future standards for assessing motor skills in children with autism in Polish conditions.

This study has several limitations that should be taken into account when interpreting the results. First, as a pilot study, it involved a relatively small sample size, which limits the generalizability of the findings. Additionally, no formal sample size calculation was performed, which may affect the statistical power of the analyses. Another limitation of this study is the use of international standardization values for the MABC-2 test. Ideally, the test should be employed in a version validated for use in Poland. However, due to the absence of such a Polish standard, the analysis was conducted using the available norms in both the ASD and control groups. It is highly probable that applying a version standardized under Polish conditions would yield different percentile distributions of the results. Nevertheless, the potential inaccuracies resulting from the use of non-local norms affect both the study and control groups equally. In the present study, the inclusion of a control group serves to minimize this analytical bias, as the observed difference may represent a systematic error occurring across both groups. Another limitation concerns the assessment procedure itself—the absence of video recordings may have increased the risk of observational errors.

Validation of the MABC-2 test in the Polish context remains an important next step. Future studies should consider including a reliability analysis of the MABC-2 within the target population. This may involve assessing both inter-rater reliability and test-retest reliability to examine the consistency of scores over time. Such analyses would provide additional insights into the quality of measurement and enhance the credibility of the findings in the context of the specific study group. Additionally, using video recordings during assessments could enhance accuracy and allow for more detailed error analysis.

## 5. Conclusions

Children with ASD without co-occurring conditions demonstrate lower levels of manual dexterity, balance and eye–hand coordination compared to neurotypical peers. If these findings are confirmed in larger samples, motor coordination disorder could be recognized as a characteristic phenotype of children with ASD. The MABC-2 Test, along with the MABC-2 Checklist, shows high comparability to the DCDQ questionnaire, the current gold standard for diagnosing developmental coordination disorder, while also providing additional insights not captured by the DCDQ. This makes it a valuable and user-friendly tool for assessing coordination in children with ASD. However, while the MABC-2 Checklist enhances the MABC-2 motor test, it should not be used as a standalone diagnostic instrument.

## Figures and Tables

**Table 1:** Standard scores achieved in the MABC-2 test by the ASD group and the control group, with children’s sex type factored in.

Component	Sex/Total	ASD Group (n = 71; 100%)	Control Group (n = 95; 100%)	Group Comparison Test Result	Effect Size ES
MD Standard Score Mdn; (Q_1_; Q_3_)	Female	9.0; (7.0; 11.0)	12.0; (10.5; 14.0)	*p* < 0.00001/#	r_G_ = 0.66
	Male	7.0; (5.0; 10.0)	12.0; (10.0; 13.0)	*p* < 0.00001/#	r_G_ = 0.68
	Test Result of Comparing Results for Sex Type in Group	NS (*p* = 0.10)/#	NS (*p* = 0.22)/#	---	
	Total	8.0; (5.0; 10.0)	12.0; (10.0; 14.0)	*p* < 0.00001/#	r_G_ = 0.68
AC Standard Score Mdn; (Q_1_; Q_3_)	Female	8.0; (5.0; 10.5)	12.0; (10.0; 14.5)	*p* < 0.00001/#	r_G_ = 0.66
	Male	9.0; (5.0; 12.0)	13.0; (12.0; 15.0)	*p* < 0.00001/#	r_G_ = 0.57
	Test Result of Comparing Results for Sex Type in Group	NS (*p* = 0.39)/#	NS (*p* = 0.10)/#	---	
	Total	8.0; (5.0; 12.0)	13.0; (11.0; 15.0)	*p* < 0.00001/#	r_G_ = 0.58
Bal Standard Score Mdn; (Q_1_; Q_3_)	Female	10.0; (7.5; 11.5)	11.0; (9.0; 14.0)	NS (*p* = 0.06)/#	
	Male	9.0; (6.0; 10.0)	10.0; (9.0; 11.0)	*p* = 0.002/#	r_G_ = 0.37
	Test Result of Comparing Results for Sex Type in Group	NS (*p* = 0.22)/#	NS (*p* = 0.12)/#	---	
	Total	9.0; (6.0; 10.0)	11.0; (9.0; 14.0)	*p* = 0.00005/#	r_G_ = 0.37
Total Result of MABC-2 Test Standard Score Mdn; (Q_1_; Q_3_)	Female	9.0; (7.0; 11.0)	13.0; (11.0; 15.0)	*p* < 0.00001/#	r_G_ = 0.75
	Male	8.0; (4.0; 10.0)	13.0; (11.0; 15.0)	*p* < 0.00001/#	r_G_ = 0.70
	Test Result of Comparing Results for Sex Type in Group	NS (*p* = 0.33)/#	NS (*p* = 0.67)/#	---	
	Total	8.0; (5.0; 11.0)	13.0; (11.0; 15.0)	*p* < 0.00001/#	r_G_ = 0.73

Annotation: MD—manual dexterity; AC—aiming and catching; Bal—balance; Mdn—median: Q_1_, Q_3_—1st and 3rd quartile; NS—not statistically significant at the level of significance *p* = 0.05; /#—Mann–Whitney U Test; r_G_—glass rank biserial correlation coefficient.

**Table 2:** Standard scores achieved in MABC-2 test by the ASD Group and the control group, with children’s age factored in.

Component	Age Group	ASD Group (n = 71; 100%)	Control Group (n = 95; 100%)	Group Comparison Test Result	Effect size ES
MD Standard Score Mdn; (Q_1_; Q_3_)	I (5–6 years old)	5.0; (4.0; 8.0)	12.0; (11.0; 15.5)	*p* = 0.0003/#	r_G_ = 0.84
	II (7–10 years old)	9.0; (6.0; 11.0)	12.0; (10.0; 14.0)	*p* < 0.00001/#	r_G_ = 0.56
	III (11–12 years old)	8.0; (6.0; 9.0)	12.0; (10.5; 14.0)	*p* < 0.00001/#	r_G_ = 0.86
	Test Result of Comparing Results in Group	NS (*p* = 0.15)/&	NS (*p* = 0.43)/&	---	
AC Standard Score Mdn; (Q_1_; Q_3_)	I (5–6 years old)	11.0; (6.0; 13.0)	12.5; (12.0; 13.5)	*p* = 0.05/#	r_G_ = 0.46
	II (7–10 years old)	8.0; (5.0; 12.0)	13.0; (10.0; 15.0)	*p* < 0.00001/#	r_G_ = 0.54
	III (11–12 years old)	9.0; (7.0; 11.0)	15.0; (13.0; 15.5)	*P* < 0.00001/#	r_G_ = 0.85
	Test Result of Comparing Results in Group	NS (*p* = 0.28)/&	*p* = 0.01/& ES: ε^2^ = 0,09	---	
Bal Standard Score Mdn; (Q_1_; Q_3_)	I (5–6 years old)	8.0; (6.0; 10.0)	11.0; (9.5; 14.0)	*p* = 0.004/#	r_G_ = 0.68
	II (7–10 years old)	9.5; (8.0; 11.0)	11.0; (9.0; 14.0)	*p* = 0.003/#	r_G_ = 0.34
	III (11–12 years old)	8.5; (6.0; 10.0)	9.0; (9.0; 9.5)	NS (*p* = 0.17)/#	---
	Test Result of Comparing Results in Group	NS (*p* = 0.28)/&	*p* = 0.001/& ES: ε^2^ = 0.14	---	
Total Result of MABC-2 Test Standard Score Mdn; (Q_1_; Q_3_)	I (5–6 years old)	8.0; (4.0; 9.0)	13.0; (12.0; 15.0)	*p* = 0.00005/#	r_G_ = 0.94
	II (7–10 years old)	8.5; (6.0; 11.0)	13.0; (11.0; 15.0)	*p* < 0.00001/#	r_G_ = 0.61
	III (11–12 years old)	7.5; (4.0; 10.0)	13.0; (12.0; 14.0)	*p* < 0.00001/#	r_G_ = 0.84
	Test Result of Comparing Results in Group	NS (*p* = 0.51)/&	NS (*p* = 0.48)/&	---	

Annotation: MD—manual dexterity; AC—aiming and catching; Bal—balance; Mdn—median: Q_1_, Q_3_—1st and 3rd quartile; NS—not statistically significant at the level of significance *p* = 0.05; /#—Mann–Whitney U Test; /&—Kruskal–Wallis Test; r_G_—glass rank biserial correlation coefficient; ε^2^—epsilon square coefficient.

**Table 3:** The occurrence of additional symptoms that may affect the motor sphere in children with the suspicion or the diagnosis of the coordination disorder.

Symptom	MABC-2 Test (n = 23; 100%)	MABC-2 Questionnaire (n = 92; 100%)	DCDQ Questionnaire (n = 56; 100%)
C.1. Disorganization	15 (65.22%)	54 (58.70%)	35 (62.50%)
C.2. Forgetfulness	19 (82.61%)	72 (78.26%)	49 (87.50%)
C.3. Inactivity	14 (60.87%)	37 (40.22%)	28 (50.00%)
C.4. Timidness	8 (34.78%)	38 (41.30%)	28 (50.00%)
C.5. Anxiety	12 (52.17%)	54 (58.70%)	38 (67.86%)
C.6. Impulsiveness	17 (73.91%)	68 (73.91%)	42 (75.00%)
C.7. Inattentiveness	21 (91.30%)	75 (81.52%)	48 (85.71%)
C.8. Hyperactivity	18 (78.26%)	63 (68.48%)	39 (69.64%)
C.9. Overestimating own capabilities	12 (52.17%)	38 (41.30%)	23 (41.07%)
C.10. Underestimating own capabilities	17 (73.91%)	60 (65.22%)	36 (64.29%)
C.11. Lack of persistence	20 (86.96%)	68 (71.74%)	45 (80.36%)
C.12. Frustration with failure	16 (69.57%)	64 (69.57%)	40 (71.43%)
C.13. Lack of satisfaction with success	7 (30.43%)	17 (18.48%)	14 (25.00%)

## Data Availability

The data that support the findings of this study are available from the corresponding author upon reasonable request.

## Abbreviations

ASD	Autism Spectrum Disorders
ADHD	Attention Deficit Hyperactivity Disorder
SI	Sensory Integration
DCD	Developmental Coordination Disorder
DCDQ	Developmental Coordination Disorder Questionnaire
MABC-2	Movement Assessment Battery for Children–second edition
MD	manual dexterity
AC	aiming and catching
BAL	balance
IQ	intelligence quotient
BMI	body mass index
